# Elements of Chemistry, Including the Most Recent Discoveries and Applications of the Science to Medicine and Pharmacy, and to the Arts

**Published:** 1841-04

**Authors:** 


Art. XI.—*Elements of Chemistry, including the most recent discoveries and applications of the Science to Medicine and Pharmacy, and to the Arts.* By Robert Kane, m.d., m.r.i.a. &c. Part I.—*Dublin,* 1840. pp. 356.

At a time when so many systematic treatises on this science are before the public, many of them fresh from the laboratories of the most distinguished cultivators of its ample domain, the appearance of a new one may seem uncalled for; yet we are glad to say, that there is a peculiar character about Dr. Kane's Elements, which disposes us to recommend it to our younger friends as perhaps the best guide we can indicate for the prosecution of their chemical studies. The objects which the author has in view may be best stated in his own words: "to present to the student an account of the general principles and facts of chemistry, and of its applications to pharmacy, to medicine and to the useful arts." Keeping this steadily in view, he intends to pass over, very briefly, when treating of the chemistry of inorganic substances, the history of those numerous bodies which, from their rarity, are objects only of scientific curiosity; dwelling rather on the properties and mode of preparation of those which are of real scientific interest, or of importance in medicine and the arts. In like manner, in the department of organic chemistry, the history will be fully discussed of all such bodies as are of importance, from their bearing upon general principles or existing theories, from their use in medicine and pharmacy, their employment in the arts or in ordinary life; whilst the numerous series of bodies which are every day discovered in organic chemistry, but which do not come under the above heads, will be dismissed with only a notice of their existence. The relations of chemical action to the functions of organized matter, the applications of chemistry to physiology and pathology, will be treated of so far as our accurate knowledge extends.

It is evident, then, that the plan of this work renders it much more suitable for the medical student than any yet within his reach; since it will communicate to him a full knowledge of general principles illustrated by facts of a character peculiarly interesting and useful to him. It only remains for us, therefore, to speak of its execution; of which, so far as the portion before us enables us to judge, it would be difficult for us to express ourselves in too high terms. This part comprehends an outline of the sciences of heat, light, and electricty, especially as related to chemical phenomena; and we have been particularly struck with the completeness of the view which is presented within a small compass, not only of the elementary principles and facts of these sciences, but also of some of their most complex phenomena, which are explained with a degree of lucid conciseness that is not often to be met with. The sections on the polarization of light and heat may be particularly instanced; the general phenomena of these two subjects, which are usually regarded as too intricate for ordinary comprehension, being so clearly explained as to be intelligible to every mind of common ability; and several interesting practical applications of principles, whose sphere seems far removed from the ordinary concerns of life, being pointed out. Throughout, we are struck with the very judicious selection which Dr. Kane has made of the scientific novelties that are constantly crowding upon the attention of the physical philosopher; and those which he has introduced are so judiciously combined with the knowledge previously accessible in elementary works, that the treatise may be regarded as truly belonging to the present era, and not formed (as too many such treatises are) upon the model of those which the rapid advance of science has caused to be antiquated within even a few years. Did our space permit, and were it easy to make a selection, we should have been glad to present our readers with a specimen or two of Dr. Kane's style; but we must content ourselves with expressing the hope that the future parts will not fall short of the promise held out by the present one; and with earnestly repeating the recommendation of the work to our readers, as the one best adapted to give them a luminous view of the present state of the principles of the science, with those applications of them which are most important to the medical man.

